# Effects of Dietary Ginsenoside Rg1 Supplementation on Growth Performance, Gut Health, and Serum Immunity in Broiler Chickens

**DOI:** 10.3389/fnut.2021.705279

**Published:** 2021-11-29

**Authors:** Zehe Song, Kaihuan Xie, Yunlu Zhang, Qian Xie, Xi He, Haihan Zhang

**Affiliations:** ^1^College of Animal Science and Technology, Hunan Agricultural University, Changsha, China; ^2^Ministry of Education Engineering Research Center of Feed Safety and Efficient Use, Changsha, China; ^3^Hunan Engineering Research Center of Poultry Production Safety, Changsha, China; ^4^Hunan Co-Innovation Center of Animal Production Safety, Changsha, China

**Keywords:** ginsenoside Rg1, growth performance, immune function, gut microbiota, broiler

## Abstract

The restriction and banning of antibiotics in farm animal feed has led to a search for promising substitutes for antibiotics to promote growth and maintain health for livestock and poultry. Ginsenoside Rg1, which is one of the most effective bioactive components in ginseng, has been reported to have great potential to improve the anti-inflammatory and anti-oxidative status of animals. In this study, 360 Chinese indigenous broiler chickens with close initial body weight were divided into 5 groups. Each group contained 6 replicates and each replicate had 12 birds. The experimental groups were: the control group, fed with the basal diet; the antibiotic group, fed basal diet + 300 mg/kg 15% chlortetracycline; and three Rg1 supplementation groups, fed with basal diet + 100, 200, and 300 mg/kg ginsenoside Rg1, respectively. The growth performance, immune function, and intestinal health of birds were examined at early (day 1–28) and late (day 29–51) stages. Our results showed that dietary supplementation of 300 mg/kg ginsenoside Rg1 significantly improved the growth performance for broilers, particularly at the late stage, including an increase in final body weight and decrease of feed conversion ratio (*P* < 0.05). Additionally, the integrity of intestinal morphology (Villus height, Crypt depth, and Villus height/Crypt depth) and tight junction (ZO-1 and Occludin), and the secretion of sIgA in the intestine were enhanced by the supplementation of Rg1 in chicken diet (*P* < 0.05). The immune organ index showed that the weight of the thymus, spleen, and bursa was significantly increased at the early stage in ginsenoside Rg1 supplementation groups (*P* < 0.05). Our findings might demonstrate that ginsenoside Rg1 could serve as a promising antibiotic alternative to improve the growth performance and gut health for broiler chickens mainly through its amelioration of inflammatory and oxidative activities.

## Introduction

Chickens in the early stage of growth have a higher incidence to be infected with disease due to their weak physiological state, underdeveloped organs, and poor immune function. Due to their excellent therapeutic effects and growth promotion properties, antibiotics have been widely used in animal formula to improve the growth performance and health of livestock and poultry (1, 2). However, the abuse of antibiotics has resulted in the crisis of public biosafety, including food safety, caused by antibiotic residues in animal products and the outbreak of antibiotic-resistant microbes (3). Therefore, seeking sustainable and biosafe alternatives to substitute antibiotics in animal feed has gained enormous interest. Plant extracts, bioreactive enzymes, and probiotics were found to have the potential to prompt the efficiency of animal production, balance the gut microbiota, and maintain body homeostasis and health (4–6).

Ginseng is a widely known and valuable plant used for different pharmacological activities worldwide and belongs to genus *Panax*, which has 13 known species and is widely distributed in many countries including in China, Korea, Japan, Russia, and North America (7, 8). Ginsenosides, also known as saponins, are the main components that exert the pharmacological action of ginseng. Rg1 is the most abundant steroid saponin, which shows the superior biomedical functions of anti-inflammation and anti-oxidation with few side effects compared with other identified ginsenosides (9).

**Figure 1:**
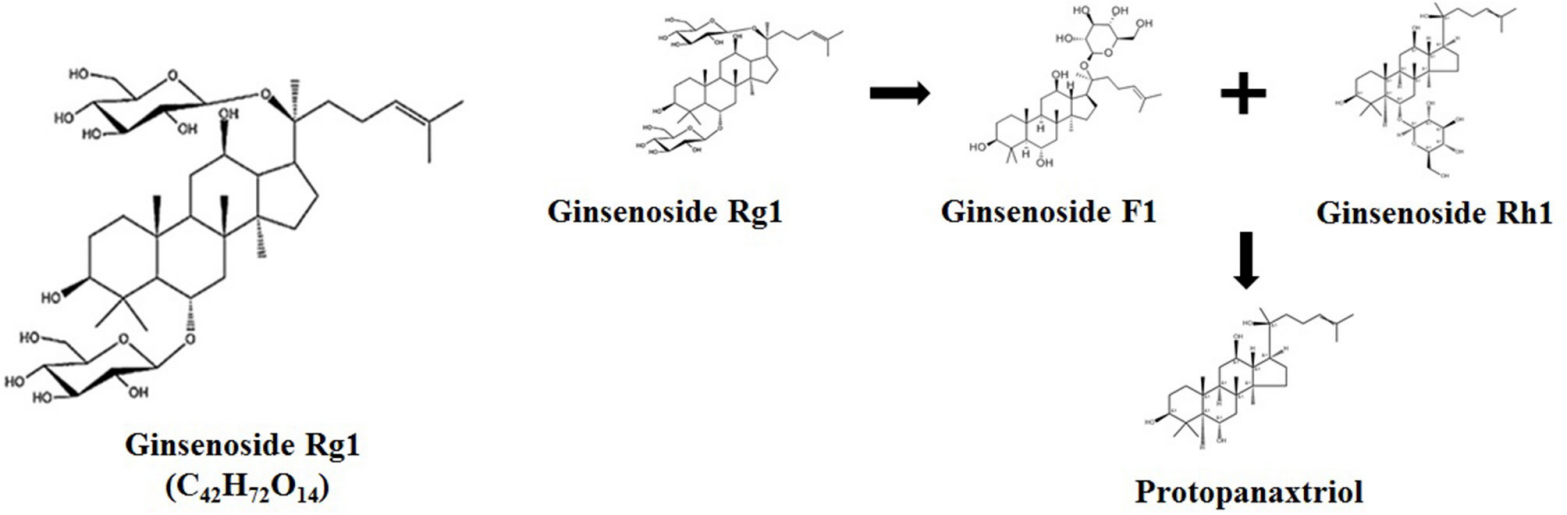
Chemical structure of Rg1 (C42H72O14) and its potential metabolic pathway.

In actual production, growers often only pick, store, and sell the roots of ginseng when it is mature, but discard the stems, leaves, and flowers that are enriched with Rg1 and still have large pharmaceutical availability. Additionally, the total content of ginsenosides Rg1 in ginseng stems and leaves is actually higher than those in ginseng roots, and the price of ginseng stems and leaves is relatively lower. Thus, the development of ginseng stem and leaf extract has great economic advantages in livestock and poultry production (10). Sandner et al. found that dietary supplementation of 90 mg/kg ginseng extract, which contained 80% of ginsenosides, significantly decreased the feed conversion ratio of broilers under heat stress (11). Additionally, 90 μg/ml ginsenoside Rg1 was able to protect chicken lymphocytes against hydrogen peroxide induced cell damage through modulating gene expression of Toll-like receptors (12). The oral administration of 1 mg/kg body weight ginsenoside Rg1 for broiler chickens showed to attenuate the immune response and oxidation stress from disease infection (13). It was reported that the ginsenoside Rg1 was metabolized into ginsenoside F1 or Rh1 and further hydrolyzed into protopanaxtriol by microbes in the intestine [Figure 1; (7, 8)].

Although many pieces of evidence have proved that ginsenoside Rg1 was able to ameliorate and protect from aging-related brain damage, attenuate the oxidative stress in liver steatosis, and reduce the plasmatic triglyceride and cholesterol level to lower the risk of obesity in model animals (14–16), little is known about the dietary effects of ginsenoside Rg1 for farm animals. Therefore, in this study, we investigated the effects of ginsenoside Rg1 supplemented in broiler chicken diets on the growth performance, carcass traits, serum immunoglobulin, immune organ index, intestinal barrier function, and intestinal morphology.

## Materials and Methods

### Reagents, Animals, and Experimental Design

A commercial ginsenoside Rg1 (Purity 90%) product was purchased from Nanjing Chunqiu biological engineering company.

Three hundred sixty 1-day-old Chinese local yellow-feathered male chickens with uniform body weight and in good health condition were obtained from Jitai animal husbandry company (Zhuzhou, Hunan). The marketing age of this Chinese local chicken breed is 56 days and the final body weight is expected to be 2.2 kg with the feed conversion ratio approximately at 2.3. A single-factor test design was performed to allocate all chickens into 5 treatment groups according to the same average body weight principle. Each treatment group consisted of 6 replicates with 12 chicks per replicate (12 birds/0.96 m^2^). The control group (CON) was fed with basal diet; the antibiotic group (ATB) was fed with basal diet + 300 mg/kg 15% antibiotic (chlortetracycline); and three Rg1 groups were fed with the basal diet supplemented with 100 (GS100), 200 (GS200), and 300 (GS300) mg/kg ginsenoside Rg1, respectively. Chickens were housed with a 23:1 h light/dark cycle, the temperature was kept between 23–27°C, and the humidity was maintained around 50~65%. Each group of birds was kept in a separate rearing isolator. Each isolator contained a wire mesh floor and was equipped with two nipple drinkers and one feeder. Both diets and water were supplied *ad libitum*. All experimental birds were husbanded for 51 days.

### Sample Collection

The body weight was recorded at day 28 and day 51, respectively, to evaluate average daily gain (ADG). Feed consumption was recorded once weekly to calculate average daily food intake (ADFI) and the feed-to-gain ratio (F/G) was calculated accordingly.

Blood samples of six birds per treatment (1 bird per pen) at days 28 and 51 were taken from the wing vein and centrifuged at 3,000 r/min for 15 min at 4°C to obtain the serums. Tissue samples including the jejunum and rectum were dissected and collected. The cecum chyme samples were collected and transferred into sterile precooled tubes, and then stored at −80°C. All procedures were approved by the Institutional Animal Care and Use Committee of Hunan Agricultural University.

### Formulation of Basal Diets

The basal diet is prepared according to nutrient requirements of poultry revised by NRC (National Research Council, US), 1994 and Chicken Feeding Standard (NY/T33-2004), and its composition and nutrition levels are shown in Table 1.

**Table 1:** Composition and nutrient levels of basal diets.

**Items**	**1–28 day**	**29–56 day**
**Diet composition, %**		
Corn	65.80	66.30
Wheat bran	0.60	1.00
Soybean meal	25.90	26.90
Fish meal	2.00	0
Soybean oil	1.70	1.80
Premix^a^	4.00	4.00
Total	100.00	100.00
**Nutrient levels** ^**b**^		
ME MJ/kg	12.88	12.32
CP, %	18.66	17.19
Lys, %	0.99	0.88
Met, %	0.27	0.26
Ca, %	0.90	0.90
TP, %	0.42	0.36

a *Premixes provided per kg of feed: Vitamin A12000 IU, Vitamin D322500 IU, Vitamin E20.0 mg, Vitamin K33.0 mg, Vitamin B13.0 mg, Vitamin B28.0 mg, Vitamin B6 7.0 mg, Vitamin B120.03 mg, Pantothenic acid 20.0 mg, Niacin 50.0 mg, biotin 0.1 mg, folic acid 1.5 mg, Fe 96 mg, Cu 25 mg, I 0.9 mg, Zn 98 mg, Mn 105.4 mg, and Se 0.04 mg*.

b *Nutrient levels are calculated values*.

### Growth Performance, Carcass Traits, and Immune Organ Index

The average weight, average daily gain (ADG), average daily feed intake (ADFI), and feed /gain (F/G) were calculated for each repetition at day 29 and day 52, respectively, and the number of deaths was recorded. On days 29 and 52, one healthy bird was randomly selected from each group per replicate, weighed, and slaughtered. The slaughter rate, full evisceration rate, abdominal fat rate, pectoral muscle rate, and leg muscle rate were measured and calculated following the equations below.

Carcass yield = (carcass weight/live weight) × 100%.
Full evisceration rate = (full net rifling weight/live weight) × 100%.
Abdominal fat yield = (abdominal fat weight/full net body weight) × 100%.
Breast yield = (breast muscle weight/full net body weight) × 100%
Leg yield = (legs muscle weight /full net body weight) × 100%.

The thymus gland, spleen, and bursa were dissected from the birds with the surrounding fat removed and weighed to calculate the immune organ index.

Organ index = organ weight (g)/live body weight (kg).

#### Meat Quality

The pH value of the thigh muscle was measured using a pH meter (Mettler Toledo, Zurich, Switzerland) after 24 h of 4°C storage. Other meat quality indexes were was determined within 45 min after euthanasia. The luminance (L^*^), redness (a^*^), and yellowness (b^*^) of the thigh muscle were measured using a colorimeter (Minolta, Tokyo, Japan). The shear force was measured using a digital tenderness meter (C-LM3B, Tenovo, Beijing, China). The drip loss was measured using a pressure gravimetric method. The initial weight of the muscle sample was first determined. Then, the sample was placed between 18 layers of filter paper in a compressor and pressed with a pressure of 2,000 psi for 1 min (17). This meat sample was reweighed immediately (final weight), and the drip loss (%) was calculated as follows:

Drip loss (%) = (initial weight-final weight)/initial weight × 100%.

### Immune Factors in Intestine and Serum

Immunoglobulins A (IgA), immunoglobulins G (IgG), immunoglobulins M (IgM), tumor necrosis factor-a (TNF-a), interleukin (IL)-1β, IL-2, IL-6, IL-10 complement C3 and C4 concentrations in serum were measured by commercial ELISA kits (Beijing Sino-UK Institute of Biological Technology, Beijing, China) using the method of Grilli et al. (18). Secretory immunoglobulin A (sIgA) concentration in jejunal mucosa and rectal mucosa was measured using a commercially available chicken ELISA kit (R&D Systems, Minneapolis, MN) according to Ma et al. (19). Total protein concentration in jejunal mucosa was measured using the method described by Smith et al. (20). Values in jejunal mucosa were expressed as units/g protein.

### Intestinal Mucosal Morphology and Tight Junction Analysis

Paraffin blocks of chicken jejunum were prepared and cut into histological sections for H&E staining. The intestinal epithelial structures were obtained by randomly selecting six places of the intestinal sections with the same magnifications and the intestinal villus height and crypt depth, and their ratio was measured by using ImageJ software (National Institutes of Health, US). Expression of zonula occludins-1 (ZO-1), claudin-1, and occludin genes in jejunal mucosa was determined by real-time qPCR. Total RNA was extracted from jejunal mucosal samples using Trizol reagent (Invitrogen, Carlsbad, CA). The quality and quantity of RNA were determined using a spectrophotometer (NanoDrop ND-1000; Thermo Fisher Scientific, Wilmington, DE). The integrity of RNA was determined by agarose gel electrophoresis. First-strand cDNA was synthesized using a reverse transcription kit (Invitrogen). Oligo 6.0 (Molecular Biology Insights, Cascade, CO) was used to design primers, which are listed in Table 2. RT-qPCR was performed with a volume of 10 μL containing 1 μL cDNA template, 5 μL SYBR Green Mix, 0.2 μL ROX Reference Dye (50 times), and 0.2 μL each of forward and reverse primers. The thermal cycling conditions were as follows: pre-denaturation (10 s at 95°C); 40 cycles of amplification (5 s at 95°C and 20 s at 60°C); and melting curve construction (60–99°C with a heating rate of 0.1°C/second and fluorescence measurements). Relative gene expression was expressed as a ratio of the target gene to the control genes using the 2^−ΔΔCt^ method.

**Table 2:** Primer sequences used for real-time quantitative PCR.

**Genes**	**Primer sequence (5^**′**^-3^**′**^)**
β-Actin	F: GAGAAATTGTGCGTGACATCA
	R: CCTGAACCTCTCATTGCCA
ZO-1	F: GCGCCTCCCTATGAGGAGCA
	R: CAAATCGGGGTTGTGCCGGA
Occludin	F: CCGTAACCCCGAGTTGGAT
	R: ATTGAGGCGGTCGTTGATG
Claudin-1	F: GCAGATCCAGTGCAAGGTGTA
	R: CACTTCATGCCCGTCACAG

### Cecal Microbial Composition 16S Sequencing

Total microbial DNA was extracted from cecal digesta using the Stool DNA Extraction Kit (Omega Biotek, Norcross, GA) according to the manufacturer's instructions. The V3–V4 hypervariable regions of the bacterial 16S rRNA gene were amplified using primers F338 (50-ACTCCTACGGGAGGCAGCAG-30) and R806 (50-GGACTACHVGGG TWTCTAAT-30), which were provided by Personalbio Company (Shanghai, China). The PCR procedures were included the predenaturation at 95°C for 3 min, 27 cycles of denaturation at 95°C for 30 s, annealing at 55°C for 30 s, elongation at 72°C for 30 s, and final extension at 72°C for 10 min. Amplicons were extracted from 2% agarose gels, and purified using the AxyPrep DNA Gel Extraction Kit (Axygen Biosciences, Union City, CA) and quantified using QuantiFluor-ST (Promega Corporation, Madison, WI). Purified amplicons were pooled in equimolar concentrations and paired-end sequenced (2^*^300) on an Illumina MiSeq platform (Illumina, San Diego, CA) according to the standard protocols. Raw FASTQ files were demultiplexed, and quality-filtered using QIIME (Version 1.17; GitHub, San Francisco, CA). Operational taxonomic units were clustered with 97% similarity cutoff using UPARSE and chimeric sequences were identified and removed using UCHIME. The taxonomy of each 16S rRNA gene sequence was analyzed by RDP Classifier (http://rdp.cme.msu.edu/) against the Silva (SSU128) 16S rRNA database using a confidence threshold of 80%.

### Statistical Analysis

All data were analyzed with one-way ANOVA followed by Tukey's multiple comparison using SPSS 26.0 software (SPSS Inc., Chicago, IL, USA). The collected data were tested by means of one-way ANOVA. The inverse sine transformation was performed for the relative abundance of cecal microbiota to make the data normal distributed. Polynomial contrasts were used to test the linear and quadratic responses to the increase of the Ginsenoside Rg1 supplementation level in the diet. Data presented were shown as means ± SD and values were considered significant at *P* < 0.05. Graphs were generated using GraphPad Prism 6.0 software.

## Results

### Effects of Dietary Rg1 Supplementation on Chicken Growth Performance, Carcass Traits, and Immune Organ Index

The effects of ginsenoside Rg1 supplemented in chicken diets on the growth performance are displayed in Table 3. The results showed that Rg1 supplementation had a significant influence (*P* < 0.05) on the chicken final body weight, ADG, and F/G at full period, and the GS300 supplementation group showed the best increasing effects on the final body weight, ADG, and F/G at full period compared with the CON group or even ATB group. Although the effects of Rg1 supplementation on the growth performance were not significant during the early stage (day 1–28), the GS300 supplementation group showed the subsequent beneficial effects on the ADFI, ADG, and F/G ratio during the late period (day 29–51) (*P* < 0.05).

**Table 3:** Effects of ginsenoside Rg1 on growth performance of broiler chickens (*n* = 6).

**Items**	**Group** ^**1**^					***p*** -value^*^		
	**CON**	**ATB**	**GS100**	**GS200**	**GS300**	**T**	**L**	**Q**
Initial weight (g)	40.12 ± 0.50	40.17 ± 0.51	40.17 ± 0.51	40.01 ± 0.50	40.83 ± 1.54	0.164	0.285	0.264
Final weight (g)	2046.29 ± 0.76^b^	2101.1 ± 0.88^ab^	2065.5 ± 0.77^b^	2073.4 ± 0.61^b^	2183.3 ± 0.04^a^	0.034	0.169	0.370
**Early period (day 1–28)**								
ADFI (g)	51.03 ± 1.24	51.35 ± 1.49	51.42 ± 0.73	52.01 ± 0.37	52.71 ± 0.64	0.331	0.384	0.899
ADG (g)	28.05 ± 0.89	27.84 ± 0.40	27.83 ± 0.52	28.24 ± 0.51	28.38 ± 0.51	0.561	0.410	0.404
F/G	1.85 ± 0.04	1.83 ± 0.06	1.85 ± 0.03	1.84 ± 0.03	1.83 ± 0.03	0.864	0.251	0.776
**Late period (day 29–51)**								
ADFI (g)	129.39 ± 0.28^c^	132.34 ± 0.13^a^	129.67 ± 2.63^bc^	129.99 ± 1.53^abc^	132.14 ± 0.48^ab^	0.049	0.191	0.185
ADG (g)	53.11 ± 1.95^b^	56.07 ± 1.67^b^	54.84 ± 2.47^b^	55.06 ± 1.18^b^	59.23 ± 4.18^a^	0.006	0.002	0.148
F/G	2.41 ± 0.07^a^	2.36 ± 0.08^a^	2.39 ± 0.13^a^	2.32 ± 0.07^a^	2.19 ± 0.11^b^	0.002	0.001	0.051
**Full period (day 1–51)**								
ADFI (g)	89.22 ± 1.31	89.85 ± 0.99	90.66 ± 2.21	89.59 ± 0.77	90.98 ± 0.74	0.327	0.138	0.982
ADG (g)	39.68 ± 1.10^b^	40.10 ± 0.84^b^	40.07 ± 0.35^b^	40.49 ± 1.05^b^	42.83 ± 1.18^a^	<0.001	<0.001	0.009
F/G	2.26 ± 0.03^a^	2.24 ± 0.05^a^	2.23 ± 0.07^a^	2.21 ± 0.05^a^	2.12 ± 0.06^b^	0.001	<0.001	0.069

*ADG, average daily gain; ADFI, average daily feed intake; F/G, feed/gain ratio*.

* *Mean T, treatment; L, linear; Q, quadratic; orthogonal polynomials were used to evaluate linear and quadratic responses to the levels of GS Rg1 treatment. To test the linear and quadratic responses to increases in the Ginsenoside Rg1 level in the diet*.

a−c *Mean values with unlike letters between different groups were significantly different (P < 0.05)*.

1 *CON, basal diet; ATB, basal diet adding 300 mg/kg 15% antibiotic (chlortetracycline); GS100, GS200, and GS300 group, basal diet adding 100, 200, and 300 mg/kg GS Rg1, respectively*.

The proportions of broiler carcass, full evisceration, breast, thigh, and abdominal fat were measured at day 52 and the results were listed in Table 4. The supplementation of 200 and 300 mg/kg ginsenoside Rg1 in chicken diet significantly increased breast and thigh yield compared with the CON group (*P* < 0.05). However, the significance of Rg1 on carcass yield and full evisceration percent were not apparent, probably due to the insignificant effects of Rg1 treatments on the weight gain of visceral organs or the skeleton weight.

**Table 4:** Effects of ginsenoside Rg1 on carcass traits of broiler chickens (*n* = 6).

**Items %**	**Group**					***p*** **-value**		
	**CON**	**ATB**	**GS100**	**GS200**	**GS300**	**T**	**L**	**Q**
Carcass yield	88.19 ± 0.95	88.51 ± 0.57	88.42 ± 0.80	88.72 ± 0.72	88.74 ± 0.66	0.811	0.279	0.999
Full evisceration	71.35 ± 0.92	72.43 ± 1.10	72.46 ± 1.21	72.80 ± 0.13	72.92 ± 0.41	0.189	0.015	0.495
Breast yield	14.55 ± 0.99^b^	14.36 ± 0.85^b^	15.63 ± 0.95^ab^	15.99 ± 0.93^a^	14.66 ± 0.41^ab^	0.070	0.343	0.024
Breast percent^*^	10.91 ± 1.06	11.15 ± 0.91	10.95 ± 0.88	11.32 ± 1.04	10.95 ± 1.40	0.957	0.779	0.773
Thigh yield	21.20 ± 0.21^bc^	20.87 ± 0.60^c^	21.94 ± 0.73^ab^	22.42 ± 0.31^a^	22.01 ± 0.73^a^	0.003	0.153	0.267
Thigh percent^*^	17.41 ± 0.24	17.13 ± 0.65	17.73 ± 0.91	17.82 ± 1.05	17.78 ± 0.89	0.505	0.359	0.704
Abdominal fat yield	2.95 ± 0.51	2.57 ± 0.32	2.42 ± 0.59	2.37 ± 0.53	2.91 ± 0.84	0.508	0.891	0.123
Abdominal fat percent^*^	2.47 ± 0.53	2.52 ± 0.80	2.38 ± 0.78	2.52 ± 0.60	3.03 ± 0.81	0.543	0.225	0.210

* *Data were calculated by the tissue weight/live body weight of broiler chickens*.

a−c *Mean values with unlike letters between different groups were significantly different (P < 0.05)*.

The rapid artificial selection on the body weight has been reported to reduce the meat quality of broiler chickens (21). Thus, in order to look at the effects of Rg1 on the meat quality of broiler chickens when it significantly increased the muscle yield, we tested the muscle quality of chicken breast, particularly including the drip loss, shear force, meat pH, and meat colors (Table 5). The results showed that the Rg1 supplementation in chicken diet was able to increase the breast meat quality, which decreased the drip loss percent and shearing force of breast muscle compared to the CON group.

**Table 5:** Effects of ginsenoside Rg1 on breast meat quality of broiler chickens (*n* = 6).

**Items**		**Group**					***p*** **-value**		
		**CON**	**ATB**	**GS100**	**GS200**	**GS300**	**T**	**L**	**Q**
Drip loss %		31.86 ± 3.95^a^	25.73 ± 1.95^b^	25.51 ± 1.78^b^	28.89 ± 4.15^ab^	27.46 ± 2.84^b^	0.013	0.05	0.088
Shearing force /kgf		1.96 ± 0.10^a^	1.96 ± 0.08^a^	1.73 ± 0.14^b^	2.11 ± 0.04^a^	1.72 ± 0.13^b^	<0.001	0.048	0.961
pH (24 h)		4.67 ± 0.26	4.70 ± 0.05	4.67 ± 0.22	4.7 ± 0.14	4.74 ± 0.09	0.949	0.518	0.726
Meat Color	L	52.65 ± 2.87	52.43 ± 1.41	51.23 ± 1.50	50.89 ± 1.68	49.96 ± 1.09	0.140	0.041	0.982
	a	0.74 ± 0.35	1.21 ± 0.37	1.58 ± 0.60	1.19 ± 1.32	1.68 ± 0.48	0.163	0.055	0.511
	b	6.50 ± 1.09	6.26 ± 0.51	6.22 ± 1.04	5.72 ± 1.10	4.84 ± 1.07	0.205	0.050	0.350

a,b *Mean values with unlike letters between different groups were significantly different (P < 0.05)*.

**Figure 2:**
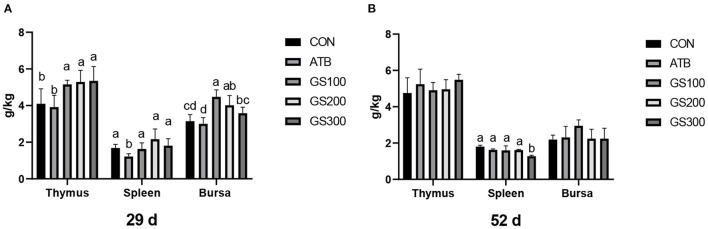
Effects of ginsenoside Rg1 on immune organ index at day 29 **(A)** and day 52 **(B)** in broiler chickens (*n* = 6). CON, birds fed a basal diet; GS100, birds fed a basal diet supplemented with 100 mg/kg ginsenoside Rg1; GS200, birds fed a basal diet supplemented with 200 mg/kg ginsenoside Rg1; GS300, birds fed a basal diet supplemented with 300 mg/kg ginsenoside Rg1; ATB, a basal diet supplemented with 300 mg/kg chlortetracycline. Data were shown as means ± standard deviations. Mean value without the common letter on the data bar in each figure indicated that the difference was significant (*P* < 0.05).

The effects of ginsenoside Rg1 on the immune organ index of broiler chickens were shown in Figure 2. On day 29, the thymus index of GS300, G200, and GS100 were significantly higher than that of the CON and ATB groups (*P* < 0.05), and showed a dose-effect. The bursa index of the G200 and GS100 groups were significantly higher than those of the CON group (*P* < 0.01). On day 52, compared with the control group, there was no significant difference in the thymus index and the bursa index of each test group. However, the Spleen index of the birds in the GS300 group was significantly lower than that of the CON group (*P* < 0.01).

### Effects of Dietary Rg1 Supplementation on Intestinal Morphology, Immunity, and Microbial Homeostasis

**Figure 3:**
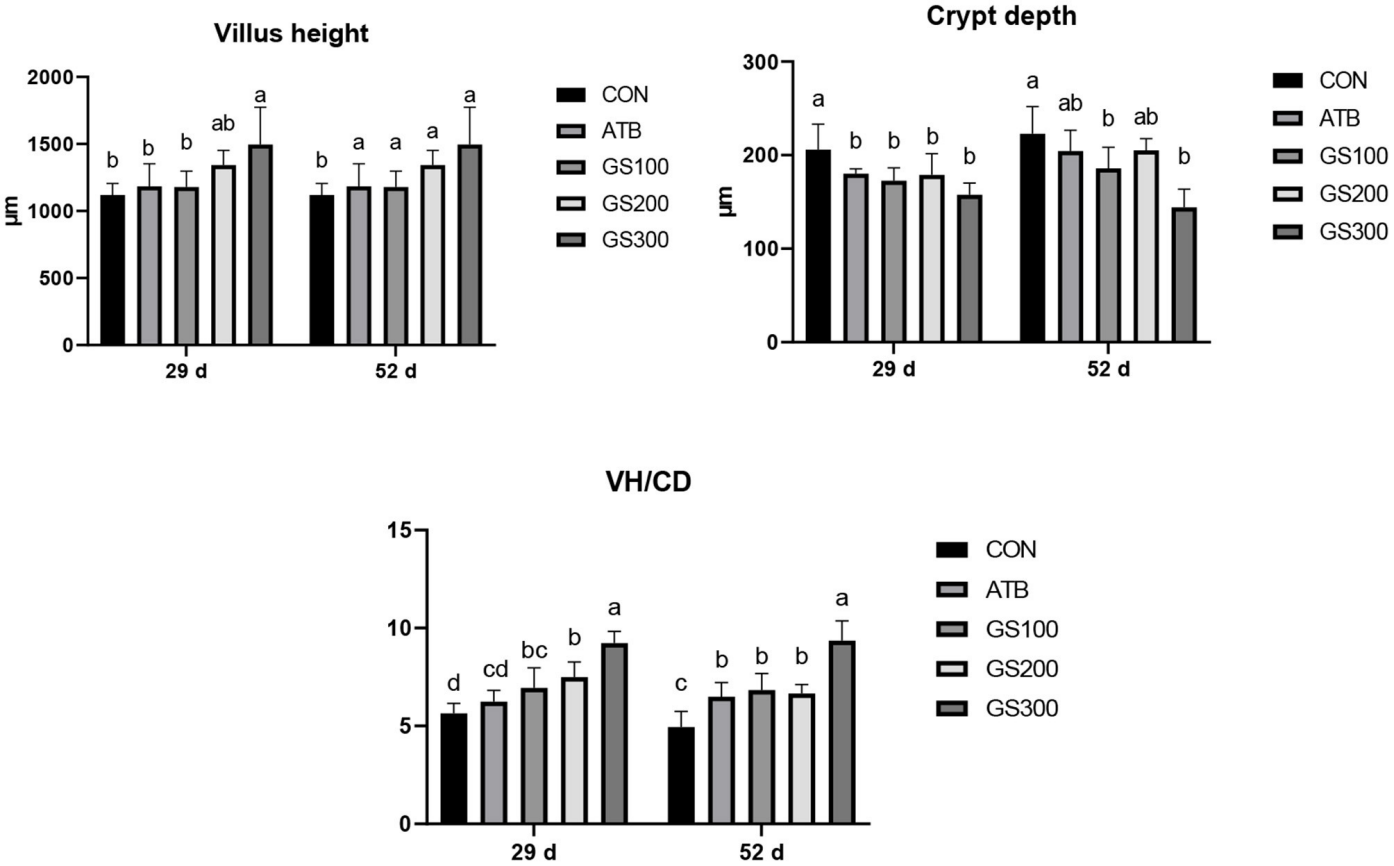
Effects of ginsenoside Rg1 on jejunal morphological structure at day 29 and day 52 in broiler chickens (*n* = 6). CON, birds fed a basal diet; GS100, birds fed a basal diet supplemented with 100 mg/kg ginsenoside Rg1; GS200, birds fed a basal diet supplemented with 200 mg/kg ginsenoside Rg1; GS300, birds fed a basal diet supplemented with 300 mg/kg ginsenoside Rg1; ATB, a basal diet supplemented with 300 mg/kg chlortetracycline. Data were shown as means ± standard deviations. Mean value without the common letter on the data bar in each figure indicated that the difference was significant (*P* < 0.05).

As shown in Figure 3, at day 29 and 52, the jejunal villus height of the birds in GS300 group was significantly higher than the CON group and ATB group. However, the crypt depth of all Rg1 supplementation groups was significantly lower than the CON group (*P* < 0.05), but not significant when compared with the ATB group. Finally, all Rg1 supplementation groups showed higher VH/CD than the CON group, and the GS300 group showed the best effects on increasing VH/CD than all other Rg1 supplementation groups, and even the ATB group.

**Figure 4:**
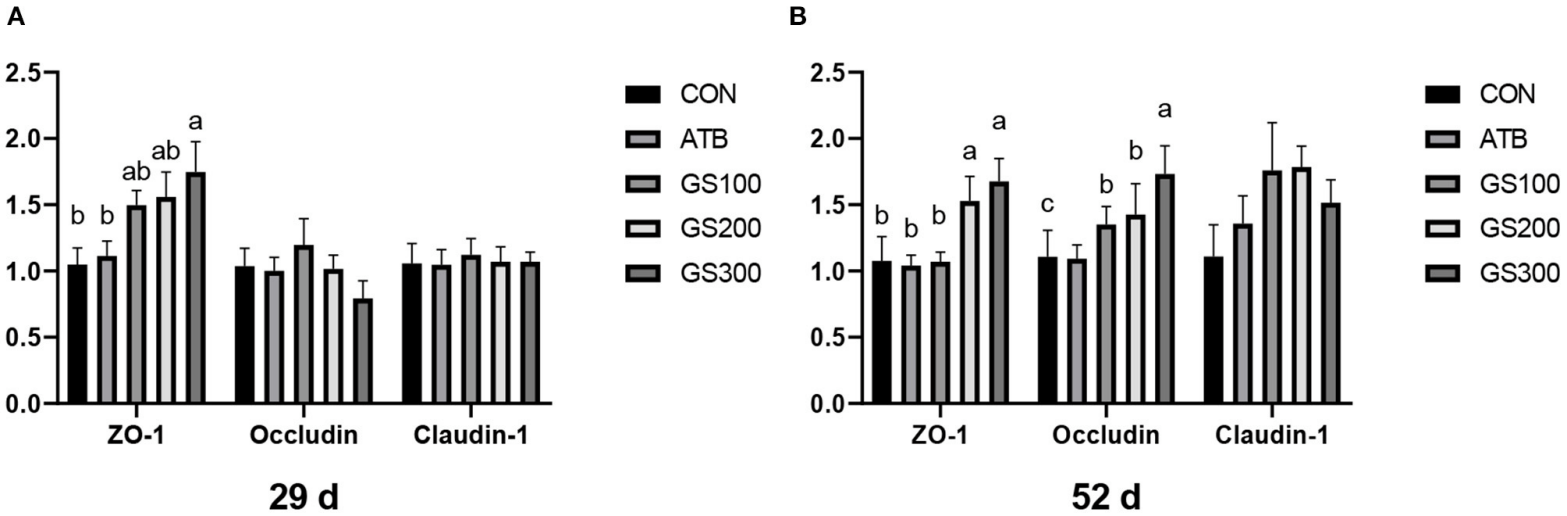
Effects of ginsenoside Rg1 on mRNA levels of key tight junction genes at day 29 **(A)** and day 52 **(B)** in broiler chickens (*n* = 6). CON, birds fed a basal diet; GS100, birds fed a basal diet supplemented with 100 mg/kg ginsenoside Rg1; GS200, birds fed a basal diet supplemented with 200 mg/kg ginsenoside Rg1; GS300, birds fed a basal diet supplemented with 300 mg/kg ginsenoside Rg1; ATB, a basal diet supplemented with 300 mg/kg chlortetracycline. Data were shown as means ± standard deviations. Mean value without the common letter on the data bar in each figure indicated that the difference was significant (*P* < 0.05).

Except for the histological observations, the integrity of intestinal epithelium is usually determined by the expression of key tight junction genes. Thus, we further measured the relative gene expression of ZO-1, Occludin, and Claudin-1 in chicken jejunum and the results were listed in Figure 4. The jejunum tight junction protein ZO-1 mRNA expression of GS300 group was significantly higher than that in the CON group at days 29 and 52, while Occludin mRNA expression in the jejunum of GS100, GS200, and GS300 group showed higher than CON group and GS300 group exhibited the greatest effect on elevating Occludin gene expression than GS100 and GS200 group at day 52.

**Figure 5:**
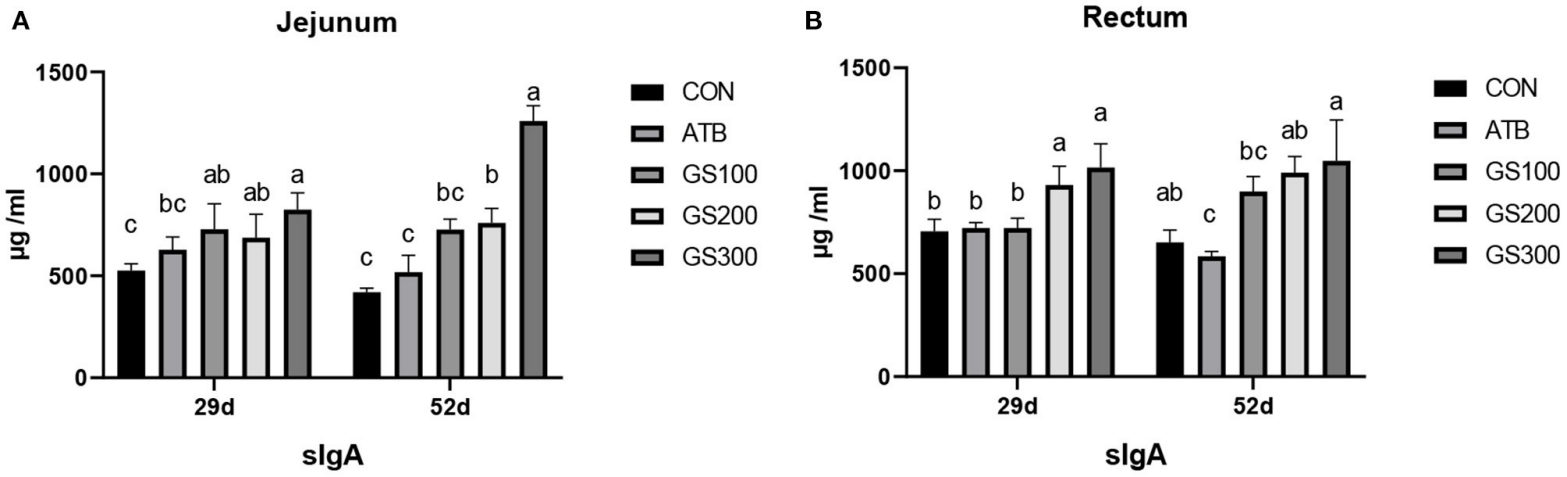
Effects of Ginsenoside Rg1 on the content of secreted Immunoglobulin A in jejunum **(A)** and rectum **(B)** in broiler chickens (*n* = 6). CON, birds fed a basal diet; GS100, birds fed a basal diet supplemented with 100 mg/kg ginsenoside Rg1; GS200, birds fed a basal diet supplemented with 200 mg/kg ginsenoside Rg1; GS300, birds fed a basal diet supplemented with 300 mg/kg ginsenoside Rg1; ATB, a basal diet supplemented with 300 mg/kg chlortetracycline. Data were shown as means ± standard deviations. Mean value without the common letter on the data bar in each figure indicated that the difference was significant (*P* < 0.05).

The immune factors that are secreted by the intestinal epithelial cells are important components constructing the intestinal chemical barrier. sIgA is the most abundant antibody which broadly distributes in the intestinal mucosa and plays a critical role in maintaining the intestinal immune homeostasis by interacting with pathogens and commensal microflora (22). As shown in Figure 5, the jejunal sIgA content of the GS300 group was significantly higher than that of the CON group and even the ATB group at days 29 and 52 (*P* < 0.01). The rectal sIgA content at days 29 and 52 increased linearly with the dose of Rg1 supplemented in the diet increased. Additionally, the sIgA contents in GS300 group was significantly higher than that of the CON group and the ATB group (*P* < 0.01) at day 29 and 52.

**Figure 6:**
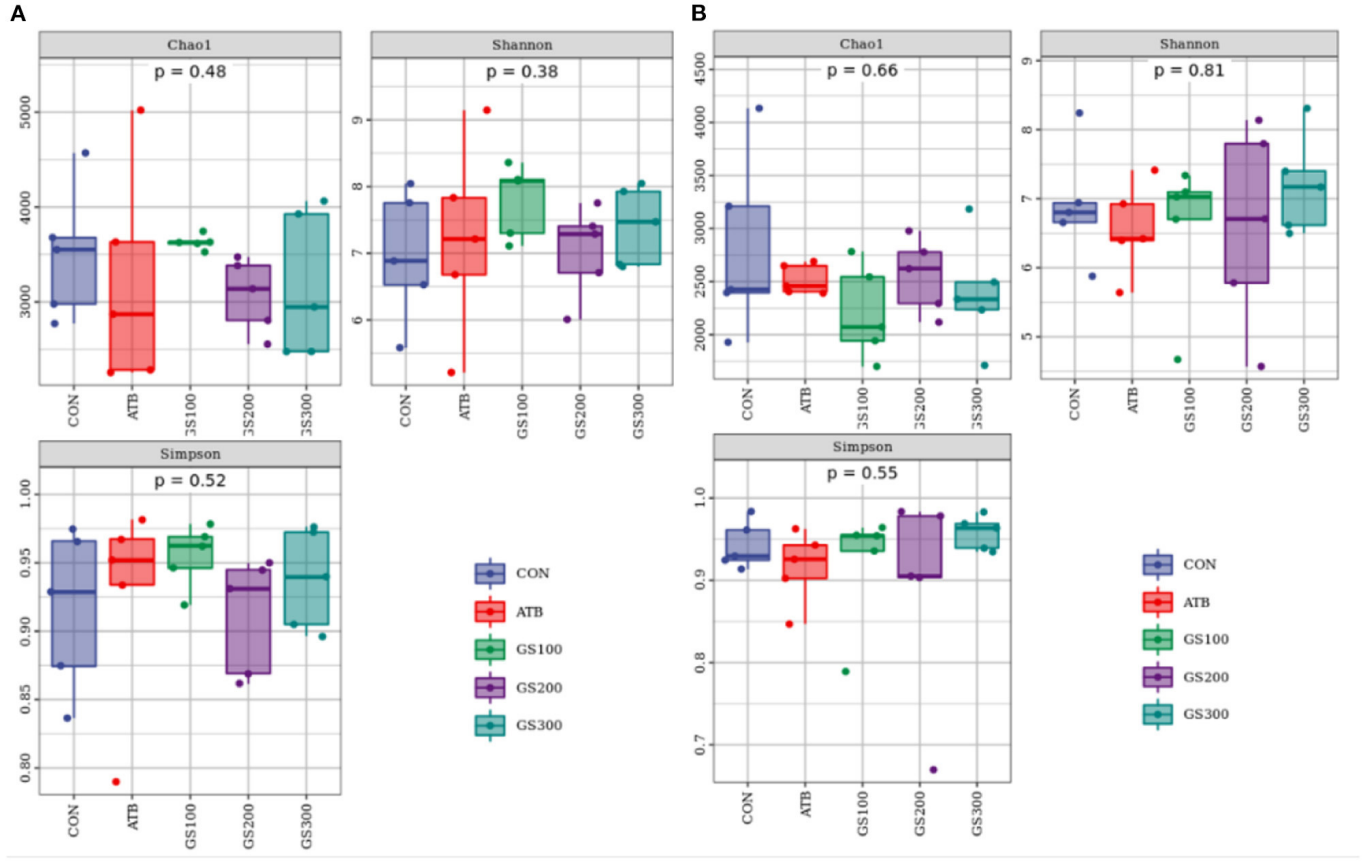
Effects of Ginsenoside Rg1 on the alpha diversity of intestinal microbiota at day 29 **(A)** and day 52 **(B)** in broiler chickens (*n* = 5). CON, birds fed a basal diet; GS100, birds fed a basal diet supplemented with 100 mg/kg ginsenoside Rg1; GS200, birds fed a basal diet supplemented with 200 mg/kg ginsenoside Rg1; GS300, birds fed a basal diet supplemented with 300 mg/kg ginsenoside Rg1; ATB, a basal diet supplemented with 300 mg/kg chlortetracycline.

Besides mechanical and chemical barriers, the intestinal microbiological barrier is a huge commensal microorganism cluster that colonizes in the mucosal layer of the intestinal epithelium and communicates with the chemical environment and mechanical activities (23). As shown in Figure 6, the alpha diversity of cecum microbiota at days 29 and 52 displayed no significant differences between different treatment groups (*P* > 0.05).

**Figure 7:**
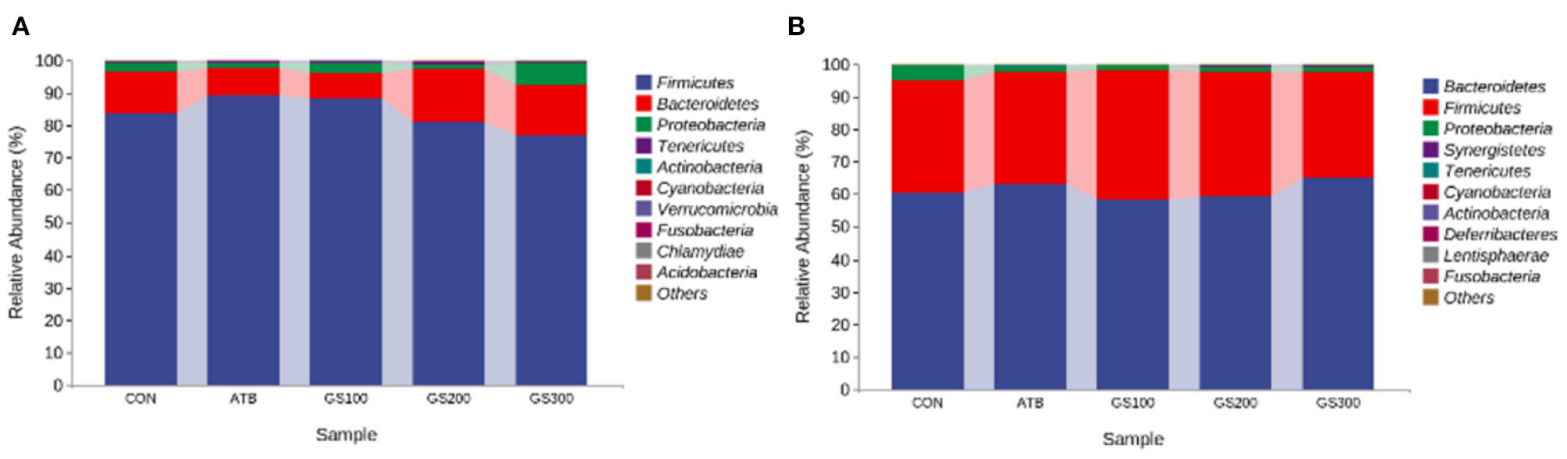
Effects of Ginsenoside Rg1 on the relative phylum abundance of intestinal microbiota at day 29 **(A)** and day 52 **(B)** in broiler chickens (*n* = 5). CON, birds fed a basal diet; GS100, birds fed a basal diet supplemented with 100 mg/kg ginsenoside Rg1; GS200, birds fed a basal diet supplemented with 200 mg/kg ginsenoside Rg1; GS300, birds fed a basal diet supplemented with 300 mg/kg ginsenoside Rg1; ATB, a basal diet supplemented with 300 mg/kg chlortetracycline.

Additionally, the taxonomic composition analysis (Figure 7) showed that Firmicutes, Bacteroidetes, and Proteobacteria were the dominant bacteria in chicken cecum both at early and late stages. But, the ratio of Firmicutes to Bacteroidetes notably decreased due to the decline of Firmicutes and increase of Bacteroidetes from day 29 to day 52 (Supplementary Tables 1, 2). Unsurprisingly, the changes of microbial composition between different treatment groups were not apparent either at the early stage or late stage, which might indicate that dietary Rg1 supplementation had little effect on the intestinal microbiota composition.

**Figure 8:**
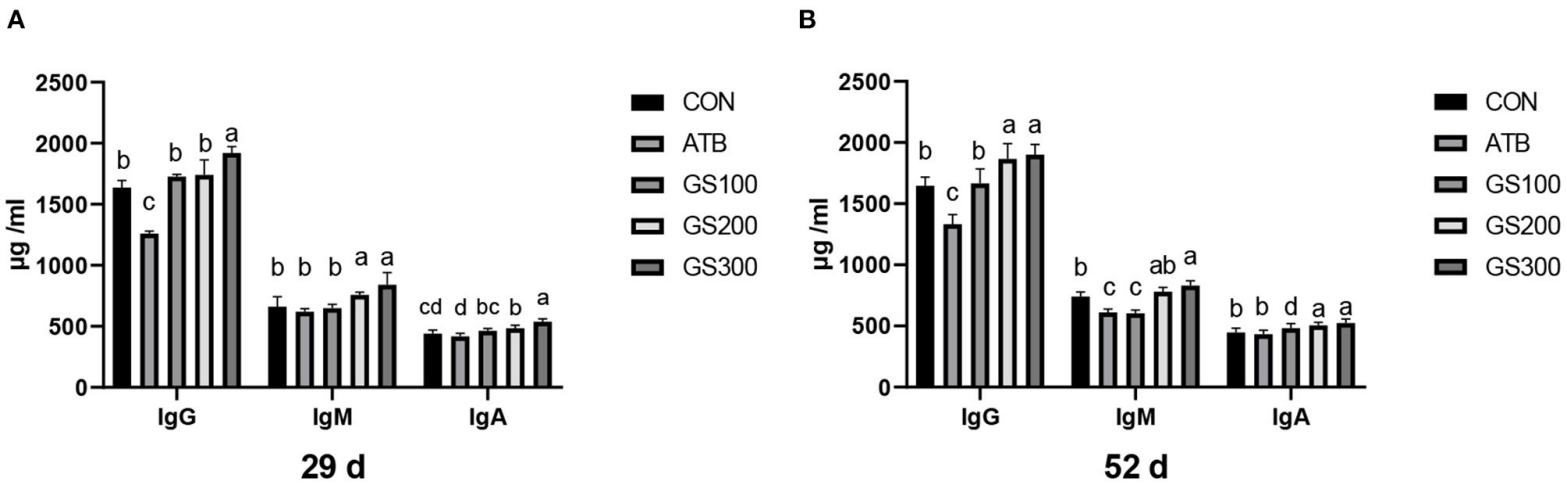
Effects of ginsenoside Rg1 on serum immunoglobulins at day 29 **(A)** and day 52 **(B)** in broiler chickens (*n* = 6). CON, birds fed a basal diet; GS100, birds fed a basal diet supplemented with 100 mg/kg ginsenoside Rg1; GS200, birds fed a basal diet supplemented with 200 mg/kg ginsenoside Rg1; GS300, birds fed a basal diet supplemented with 300 mg/kg ginsenoside Rg1; ATB, a basal diet supplemented with 300 mg/kg chlortetracycline. Data were shown as means ± standard deviations. Mean value without the common letter on the data bar in each figure indicated that the difference was significant (*P* < 0.05).

The effects of ginsenoside Rg1 on serum immunoglobulins of broiler chickens are shown in Figure 8. The increased amount of Rg1 supplemented in the diet resulted in the linear increase of IgG, IgM, and IgA in the chicken serum. Especially, on days 29 and day 52, the birds in the GS300 group had the highest IgG, IgM, and IgA contents in the serum compared with other treatment groups or the CON group (*P* < 0.01). The IgM and IgA content of all Rg1 supplementation groups were significantly higher than those in the CON and ATB groups (*P* < 0.01). On day 52, the contents of IgG and IgA in the GS300 and GS200 group were significantly higher than those in the control and antibiotic groups (*P* < 0.01), the IgM content of the GS300 group was significantly higher than that in the CON and ATB groups (*P* < 0.01), and he IgM content of the GS200 group was significantly higher than that of the ATB group (*P* < 0.01).

**Figure 9:**
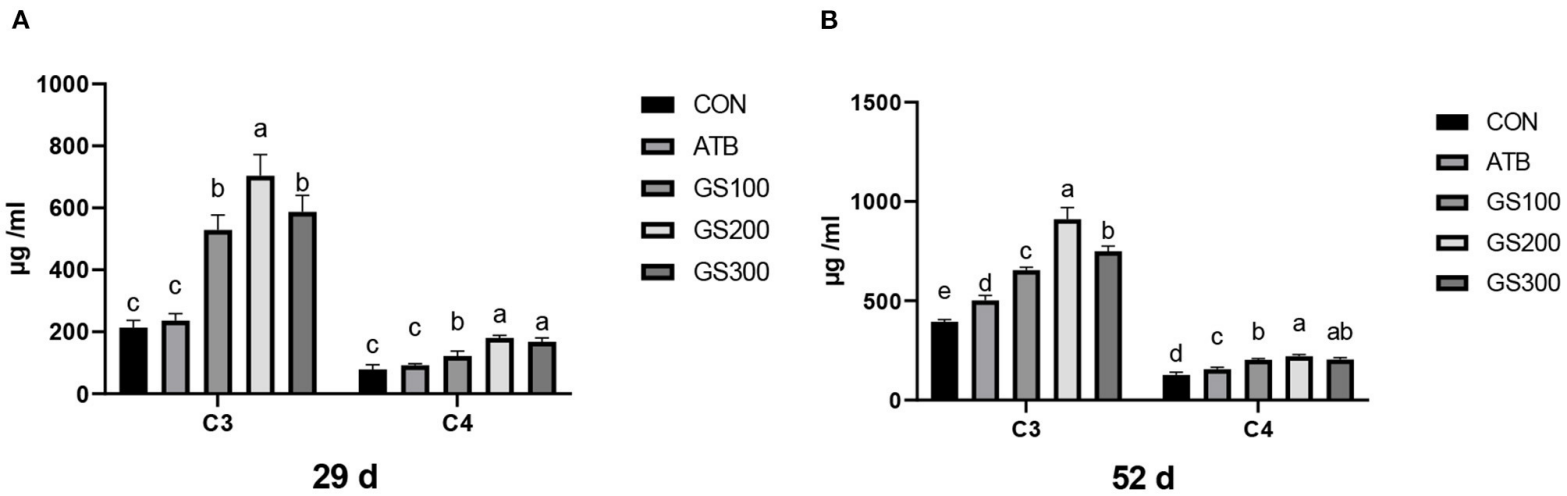
Effects of ginsenoside Rg1 on serum complement C3 and C4 at day 29 **(A)** and day 52 **(B)** in broiler chickens (*n* = 6). CON, birds fed a basal diet; GS100, birds fed a basal diet supplemented with 100 mg/kg ginsenoside Rg1; GS200, birds fed a basal diet supplemented with 200 mg/kg ginsenoside Rg1; GS300, birds fed a basal diet supplemented with 300 mg/kg ginsenoside Rg1; ATB, a basal diet supplemented with 300 mg/kg chlortetracycline. Data were shown as means ± standard deviations. Mean value without the common letter on the data bar in each figure indicated that the difference was significant (*P* < 0.05).

The effects of ginsenoside Rg1 supplementation on serum complement C3 and C4 in broiler chickens are shown in Figure 9. On days 29 and 52, the levels of serum complement C3 and C4 in the GS100, GS200, and GS300 groups were significantly higher than those of the CON group, or even ATB group (*P* < 0.01).

**Figure 10:**
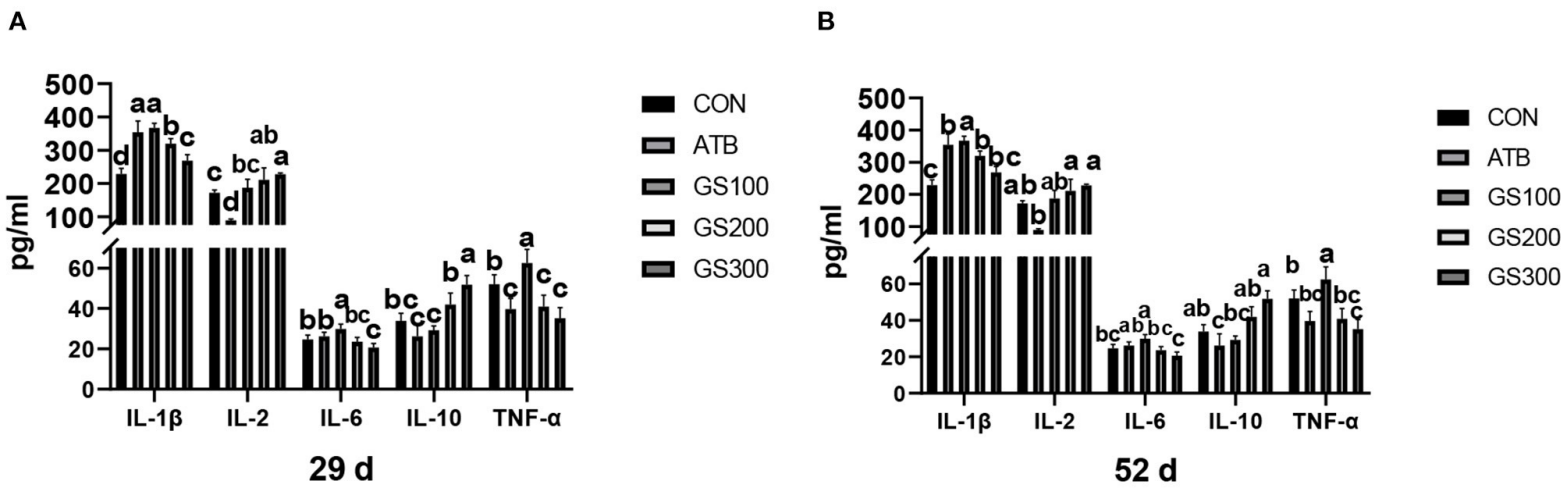
Effects of ginsenoside Rg1 on serum cytokines at day 29 **(A)** and day 52 **(B)** in broiler chickens (*n* = 6). CON, birds fed a basal diet; GS100, birds fed a basal diet supplemented with 100 mg/kg ginsenoside Rg1; GS200, birds fed a basal diet supplemented with 200 mg/kg ginsenoside Rg1; GS300, birds fed a basal diet supplemented with 300 mg/kg ginsenoside Rg1; ATB, a basal diet supplemented with 300 mg/kg chlortetracycline. Data were shown as means ± standard deviations. Mean value without the common letter on the data bar in each figure indicated that the difference was significant (*P* < 0.05).

The contents of serum cytokines are critical to indicating the inflammation status of animals. Thus, we tested the serum cytokine contents to investigate whether ginsenoside Rg1 supplementation might improve the anti-inflammatory activities for broiler chicken. The results are displayed in Figure 10. At 29 days, the IL-1β, IL-2, and IL-10 contents of the GS300 group were significantly higher than the CON group (*P* < 0.01), while the IL-6 and TNF-α contents of the GS300 group were significantly lower than CON group (*P* < 0.01). In addition, the IL-1β and IL-2 contents of the GS200 group were significantly higher than the CON group (*P* < 0.01), but the TNF-α contents of the GS200 group were significantly lower than the CON group (*P* < 0.01). At 52 days of age, the IL-1β contents of GS200 and GS100 groups were significantly higher than the CON group (*P* < 0.01), and IL-2 contents of GS300 and GS200 groups were significantly higher than the ATB group (*P* < 0.01). What is more, the IL-6 content of the GS300 group was significantly lower than the ATB group (*P* < 0.01); but the IL-10 content of the GS300 group was significantly higher than the ATB group (*P* < 0.01). Finally, the TNF-α content of the GS300 group was significantly lower than the CON group (*P* < 0.01).

## Discussion

Ginseng, as a valuable medical herbs of the past, has been now widely cultivated and grown in many countries due to its abundance of ginsenosides, which were proved to have excellent anti-inflammatory, anti-apoptotic, and anti-oxidative effects in animals (8). One of the most prominent functions of ginsenoside Rg1 is to improve cardiovascular activities including activating the vascular endothelial angiogenesis (24) and protecting the oxidative attack on cardiomyocytes in humans and rats (25, 26).

In our study, the addition of ginsenoside Rg1 in the diet has no significant effect on the growth performance of broiler chickens in the early period (1–28 day), while it showed improving effects on the ADFI, ADFG, and F/G in the late period (29–51 day), which was consistent with the previous findings that dietary supplementation of ginseng extract significantly decreased the F/G and alleviated the heat stress for broilers (11). Additionally, the final yield and meat quality of breast and thigh were significantly improved with the supplementation of ginsenoside Rg1. The supplementation of red ginseng marc also showed to increase the water holding capacity and shearing force for broiler chickens (27). He et al. (28) also found that subcutaneous injection of Rg1 caused weight gain in mice. In DSS (dextran sodium sulfate)-induced mouse colitis, ginsenoside Rg1 treatment significantly ameliorated the weight loss by mitigating the inflammatory burdens (29). It has also been demonstrated that ginsenosides Rg1 enabled to improve feed intake and production performance by increasing the antioxidant capacity of the body and alleviating oxidative stress (30, 31). Due to the rapid growth rate, especially the fast meat production during the late period of broiler chickens, the oxidative and inflammatory stress caused by this intensive metabolism could be a major reason that results in muscular dysplasia (32), visceral organ inflammation (33), and bone abnormality (34), which further leads to the low growth performance for broiler chickens (21). Thus, it is possible that the supplementation of Rg1 in broiler chicken diet at the grow-out period was able to improve the growth performance and feed conversion ratio due to its potential effects on anti-inflammation and anti-oxidation in animals. Although the role of Rg1 to mitigate chicken oxidative status was not investigated in our research, further studies should be worthwhile to test the anti-oxidative function of Rg1 on broilers.

One possible reason for Rg1-induced weight gain for broilers could be its positive effects on the intestinal morphology and integrity, thus increasing the absorption of nutrients and functional molecules and avoiding the invasion of enteric pathogens. The villi height (VH) and crypt depth (CD) are important indicators to reflect the intestinal morphology and represent the ability of nutrient absorption and epithelium renewal (35). In this study, dietary ginsenoside Rg1 supplementation could significantly increase the VH and VH/CD, and decreased the CD in the chicken jejunum. Our results might indicate that the enlarged absorptive area of chicken intestine caused by Rg1 supplementation perhaps boosted the nutrient uptake and further resulted in the bodyweight accumulation. Previous reports showed that dietary supplementation of probiotics or prebiotics increased intestinal morphology significantly by reshaping the intestinal microflora for broilers (36, 37). However, our microbial results did not present any changes when supplemented with the ginsenoside Rg1 in chicken diets. The integrity of epithelial cellular junctions determines the permeability of the intestine, thus the expression of the responsible junction proteins may indicate the ability of the intestine to filter the exogenous pathogens. The 300 mg/kg Rg1 supplementation in chicken diet showed significantly higher mRNA expression of tight junction proteins such as Occluding and ZO-1 at day 52 compared to the CON group or even the ATB group. The intestine is not only the major place to metabolize and absorb the nutrients, but also the initial barrier to prevent the pathogens or toxins into the blood (38). Hence, an impaired epithelial mechanical barrier consequently causes the failure of animal growth performance (39). The ginsenoside Rg1 may play an important role in shaping the healthy epithelial barrier by inducing the gene expression of tight junction proteins and resist the invasion of external enteropathogens and endogenous inflammation or stress.

Except for the mechanical barrier, the intestinal mucosal barrier also consists of chemical barriers and microbial barriers, which play important roles to interact with the host immune system and defend exotic pathogens (40–42). Secretory IgA (sIgA) is an antibody secreted by B lymphocytes in the lamina propria in the gastrointestinal tract and presents on the mucosal surface to form an immunoprotective layer that prevents pathogens from adhering to the cell surface and invading the circulation (43). Similar to Ma et al. (19) who found that ginsenosides could enhance the immunomodulatory effects of broilers and increase the content of sIgA. In our results, the dietary ginsenoside Rg1 supplementation could also significantly increase sIgA contents in jejunum and rectum both at an early stage and late stage. Similarly, the tight junction protein ZO-1 was also highly expressed at the early stage. However, we only observed the significant weight gain at the late stage. The possible reason was that the intensive metabolism and rapid growth burden of broiler chickens at late stage produced large oxidative and inflammatory attacks, which may cause the impairment of intestinal health for birds that closed to grow out. The supplementation of Rg1 was able to balance the intestinal homeostasis through synthesizing tight junction proteins and sIgA, which primarily played their roles to ameliorate the oxidative and inflammatory stress occurred at the late stage. However, we did not observe any compositional change of chicken cecal microbiota when the ginsenoside Rg1 was supplemented in the diet in our research, although the microbial metabolites of ginsenoside Rg1 was found to interact with the bacterial community through changing the redox metabolic events in mice (44).

Interestingly, we found that the supplementation of Rg1 in the chicken diet was particularly able to increase the weight of critical immune organs, such as the thymus, spleen, and bursa of Fabricius at an early stage. Therefore, the Rg1 might play a role in facilitating the maturation of immune organs and this result stimulated us to investigate whether Rg1 supplementation could improve the immune status of the birds through generating and secreting more complement components, immunoglobulins, or cytokines in the serum. Strikingly, dietary ginsenoside Rg1 apparently elevated the anti-inflammatory cytokines including IL-10, and IL-2, and declined pro-inflammatory cytokines such as IL-6, IL-1β, and TNF-α in chicken serum. It has been reported that ginsenoside Rg1 could target the liver and decrease the production of IL-1β by suppressing the hepatic inflammation induced by nucleotide-binding oligomerization domain (NOD)-like receptor family pyrin domain-containing 3 (NLRP3) (45). In addition, the serum IL-6, IL-1β, and TNF-α in rats suffering from alcoholic hepatitis was dropped back almost to the normal level via inhibiting the activation of the NF-κB pathway when treated with ginsenoside Rg1 (46). Complement components and immunoglobulins are two important immune factors in the serum and constitute the non-specific and specific immunity, respectively. Consistent with the previous findings that Rg1 could induce the serum IgG and stimulate the synthesis of IgA in B cells in mice (47, 48), our results showed that the supplementation of 300 mg/kg ginsenoside Rg1 in broiler diet was able to increase the C3, C4, IgA, IgM, and IgG content in the serum, suggesting that Rg1 might strengthen the ability of the immune defenses for broiler chickens.

## Conclusions

In summary, the dietary supplementation of 300 mg/kg ginsenoside Rg1 was found to have great beneficial potential on growth performance for broilers, particularly at the late stage, including the increase of final body weight and decrease of feed conversion ratio. This positive effect could be associated with the function of ginsenoside Rg1 in increasing the ability to absorb nutrients and resist pathogens through improving the morphology of the intestine, integrity of tight junctions, and secretion of sIgA. Additionally, Rg1 supplementation significantly boosted the capacity of serum immunity by enhancing the anti-inflammatory and anti-oxidative abilities of the body, such as the suppression of pro-inflammatory cytokine secretion and activation of anti-inflammatory cytokines, complement system, and immunoglobulin production. Therefore, according to our study, the application of ginsenoside Rg1 in the diet could be a potential alternative with which to substitute antibiotics to improve the growth performance and body health of broiler chickens.

## Data Availability Statement

The datasets presented in this study can be found in online repositories. The names of the repository/repositories and accession number(s) can be found at: https://www.ncbi.nlm.nih.gov/, PRJNA726376.

## Ethics Statement

The animal study was reviewed and approved by Institutional Animal Care and Use Committee at Hunan Agricultural University.

## Author Contributions

ZS and KX were mainly responsible for the experimental design, animal feeding, and result analysis. YZ and QX carried out the serum biochemistry analysis and meat quality test, respectively. XH and HZ were responsible for the data summarizing, manuscript writing, modification, and submission. All authors contributed to the article and approved the submitted version.

## Funding

This study was supported by the National Key R&D Program of Intergovernmental Key Projects of China (Grant No. 2018YFE0101700); Guangxi science and technology base and talent project (AD20238092); and support project for scientific and technical talents in Hunan Province (2020TJ-Q02) and Hunan Hundred Talents Program.

## Conflict of Interest

The authors declare that the research was conducted in the absence of any commercial or financial relationships that could be construed as a potential conflict of interest.

## Publisher's Note

All claims expressed in this article are solely those of the authors and do not necessarily represent those of their affiliated organizations, or those of the publisher, the editors and the reviewers. Any product that may be evaluated in this article, or claim that may be made by its manufacturer, is not guaranteed or endorsed by the publisher.
