# Supplementary material for: Effects of Dietary Ginsenoside Rg1 Supplementation on Growth Performance, Gut Health, and Serum Immunity in Broiler Chickens
Source: Front Nutr. 2021 Nov 29;8:705279. doi: 10.3389/fnut.2021.705279 (PMC8667319; doi:10.3389/fnut.2021.705279)
Supplement: Supplementary file 1 [file Table_1.DOCX]

**Supplementary table 1. Relative abundance of top 5 phylum of cecal microbiota in broilers supplemented with ginsenoside Rg1 at day 29 (n=5).**

| Phylum | CON | ATB | GS100 | GS200 | GS300 | *P*-value |
| --- | --- | --- | --- | --- | --- | --- |
| Firmicutes | 1.159±0.073 | 1.246±0.11 | 1.23±0.093 | 1.145±0.219 | 1.092±0.189 | 0.474 |
| Bacteroidetes | 0.36±0.095 | 0.288±0.102 | 0.271±0.086 | 0.386±0.229 | 0.382±0.178 | 0.627 |
| Proteobacteria | 0.137±0.102 | 0.095±0.081 | 0.147±0.127 | 0.099±0.055 | 0.214±0.161 | 0.467 |
| Tenericutes | 0.058±0.032 | 0.065±0.035 | 0.066±0.025 | 0.084±0.044 | 0.073±0.032 | 0.789 |
| Actinobacteria | 0.023±0.011 | 0.017±0.006 | 0.014±0.005 | 0.022±0.006 | 0.026±0.012 | 0.233 |
| Firmicutes/Bacteroidetes | 3.419±0.939 | 5.001±2.5 | 5.016±1.909 | 4.203±3.06 | 3.639±2.271 | 0.703 |

Note: Data were presented as means ± standard deviations, which were calculated from the inverse sine transformation of the bacterial relative abundance.
