# Supplementary material for: Effects of Dietary Ginsenoside Rg1 Supplementation on Growth Performance, Gut Health, and Serum Immunity in Broiler Chickens
Source: Front Nutr. 2021 Nov 29;8:705279. doi: 10.3389/fnut.2021.705279 (PMC8667319; doi:10.3389/fnut.2021.705279)
Supplement: Supplementary file 2 [file Table_2.DOCX]

**Supplementary table 2. Relative abundance of top 5 phylum of cecal microbiota in broilers supplemented with ginsenoside Rg1 at day 52 (n=5).**

| Phylum | CON | TAB | CS100 | CS200 | CS300 | P-value |
| --- | --- | --- | --- | --- | --- | --- |
| Bacteroidetes | 0.897±0.126 | 0.92±0.124 | 0.873±0.211 | 0.884±0.113 | 0.941±0.143 | 0.948 |
| Firmicutes | 0.623±0.111 | 0.627±0.13 | 0.68±0.213 | 0.664±0.109 | 0.606±0.145 | 0.923 |
| Proteobacteria | 0.166±0.148 | 0.107±0.026 | 0.096±0.039 | 0.099±0.067 | 0.106±0.029 | 0.582 |
| Synergistetes | 0.000 | 0.023±0.038 | 0.008±0.019 | 0.035±0.055 | 0.035±0.05 | 0.512 |
| Tenericutes | 0.038±0.011 | 0.041±0.004 | 0.038±0.017 | 0.035±0.006 | 0.038±0.019 | 0.955 |
| Firmicutes/Bacteroidetes | 1.502±0.46 | 1.555±0.536 | 1.444±0.666 | 1.392±0.478 | 1.656±0.547 | 0.947 |

Note: Data were presented as means ± standard deviations, which were calculated from the inverse sine transformation of the bacterial relative abundance.
